# Editorial: Myocardium regeneration and cardioprotection

**DOI:** 10.3389/fmmed.2023.1293183

**Published:** 2023-10-09

**Authors:** Martina Iengo, Ester Topa, Alessandra Cuomo, Maria Cristina Luise, Francesco Fiore, Marika Rizza, Mattia Miccio, Elena Di Sarro, Giuseppe Ciaccio, Chiara Di Lorenzo, Valentina Mercurio, Sang-Bing Ong, Serena Zacchigna, Carlo Gabriele Tocchetti

**Affiliations:** ^1^ Department of Translational Medical Sciences, Federico II University, Naples, Italy; ^2^ Interdepartmental Center of Clinical and Translational Sciences (CIRCET), Federico II University, Naples, Italy; ^3^ Interdepartmental Hypertension Research Center (CIRIAPA), Federico II University, Naples, Italy; ^4^ Department of Medicine and Therapeutics, Faculty of Medicine, The Chinese University of Hong Kong, Shatin, China; ^5^ Centre for Cardiovascular Genomics and Medicine (CCGM), Lui Che Woo Institute of Innovative Medicine, Chinese University of Hong Kong (CUHK), Hong Kong SAR, China; ^6^ Hong Kong Hub of Paediatric Excellence (HK HOPE), Hong Kong Children’s Hospital (HKCH), Kowloon Bay, Hong Kong SAR, China; ^7^ Kunming Institute of Zoology–The Chinese University of Hong Kong (KIZ-CUHK) Joint Laboratory of Bioresources and Molecular Research of Common Diseases, Hong Kong SAR, China; ^8^ Neural, Vascular, and Metabolic Biology Thematic Research Program, School of Biomedical Sciences (SBS), Chinese University of Hong Kong (CUHK), Hong Kong SAR, China; ^9^ International Centre for Genetic Engineering and Biotechnology, Trieste, Italy; ^10^ Department of Medicine, Surgery and Health Sciences, University of Trieste, Trieste, Italy; ^11^ Center for Basic and Clinical Immunology Research (CISI), Federico II University, Naples, Italy

**Keywords:** myocardium regeneration, cardioprotection, stem cells, cardiomyocytes, heart failure

Failure to regenerate the heart following injury is one of the main causes of heart failure and death worldwide. Billions of cardiomyocytes usually die within a few hours after myocardial infarction and are replaced by fibrotic tissue, which is stiffer than the myocardium and with no contractile capacity.

This loss of cardiomyocytes is not accompanied by regeneration in adult mammals. Over the past 15 years, activated adult stem cells have been proposed as a potential source of new cardiomyocytes (Ciucci et al., 2022). New cardiomyocytes can also be obtained from embryonic stem (ES) cells. These new cardiomyocytes can be injected into the heart and drive partial regeneration (Ciucci et al., 2022). The concept that heart regeneration can be achieved by either implanting cardiomyocytes obtained in the laboratory from ES cells, or by stimulating the proliferative potential of cardiomyocytes, is innovative and exciting. In the case of cardiomyocytes derived from stem cells, the main problems are the limited number of cells that can be obtained, their immunogenicity and need for immunosuppression, and their incomplete maturation, which impairs their full electrical and mechanical integration and thereby can generate serious arrhythmias. In the case of cardiac regeneration obtained by stimulating the endogenous ability of cardiomyocytes to proliferate, the main problems are both efficiency of gene transfer to the myocardium and possible long-term side effects (Gabisonia et al., 2019).

During myocardium development, cardiomyocytes gradually exit from the cell cycle and become unable to proliferate after birth. Nonetheless, recent studies have shown that adult cardiomyocytes can re-enter the cell cycle and proliferate under certain mitogen stimulations. This seems to be limited to a small number of mono-nuclear cardiomyocytes. Various ncRNAs, including miRNA, lncRNA, and circRNA control the essential genes and regulatory pathways of various cellular processes, including cell proliferation in development and disease. Numerous studies have been recently published on the role of miRNAs in cardiac regeneration, although there are still significant challenges in their clinical translation (Qin et al.).

As said, cardiac fibrosis is central to both ischemic and non-ischemic heart failure (HF). Adjacent cells communicate through gap junction (GJ) channels composed of connexins, which results in the electric communication between cardiomyocytes to coordinate contraction. The heart’s three major interstitial GJ proteins are Cx40, Cx43, and Cx45. Cx43 is ubiquitously distributed in various tissues and organs of mammals, especially in the heart and one paper from this Research Topic shows that decreased Cx43 expression is associated with excessive fibrotic deposition and ventricular dysfunction (Liu et al.).

Interestingly, the crosstalk between cardiac cytotoxic memory CD8^+^ T cells and cardiomyocytes is essential for development of non-ischemic hypertensive cardiac fibrosis (Brassington et al.). Interaction between cytotoxic memory CD8^+^ T cells and overly stressed cardiomyocytes is highly dependent on the innate stress-sensing receptor NKG2D on CD8^+^ T cells and RAE-1 expressed by cardiomyocytes. Preventing CD8^+^ T cell activation by inhibiting the cardiomyocyte RAE-1-CD8^+^ T cell-NKG2D axis is presented as a promising strategy to limit hypertensive cardiac fibrosis. CD8^+^ T cells seem to recruit monocyte/macrophages and modulate their activity toward a cardioprotective phenotype to maintain homeostasis early during myocardial stress.

At the time of myocardial infarction, hyperglycemia has an adverse effect on prognosis. In this Research Topic Brennan et al. show that exposure to elevated glucose for >7.5 min decreased contractile recovery in Freshly isolated non-diabetic rat cardiomyocytes, suggesting its potential cardiotoxicity. If the duration of high glucose challenge was less than 5 min, it did not cause cardiotoxicity, but enhanced cell survival, indicating cardioprotection, analogously to brief ischaemic intervals used in ischaemic preconditioning.

Finally, in another manuscript, Kapchoup et al. assess Hydroxychloroquine (HDQ)’s cardioprotective effect, and its role as a possible candidate for tissue repair. The Authors show that HDQ (20 μM) promotes proliferation of stem cells, but also indicate that HDQ is potentially toxic at high concentrations and can modulate the beating activity of cardiomyocytes. In fact, higher doses of HDQ cause bradycardia and negative inotropic activity that can be reversed with a high concentration of the ß-adrenergic agonist Isoproterenol (Iso), and significantly induce apoptosis of cardiac muscle cells.

Collectively, the papers in this Research Topic shed light on a very intricate but fascinating Research Topic. Further investigation is required to bring these results into innovative therapies for cardioprotection and cardiac regeneration.

## References

[B1] Ciucci G. Colliva A. Vuerich R. Pompilio G. Zacchigna S. (2022). Biologics and cardiac disease: challenges and opportunities. Trends Pharmacol. Sci. 43 (11), 894–905. 10.1016/j.tips.2022.06.001 35779965

[B2] Gabisonia K. Prosdocimo G. Donato Aquaro G. Carlucci L. Zentilin L. Secco I. (2019). MicroRNA therapy stimulates uncontrolled cardiac repair after myocardial infarction in pigs. Nature 569 (7756), 418–422. 10.1038/s41586-019-1191-6 31068698 PMC6768803

